# Chicago Medical College

**Published:** 1873-09-15

**Authors:** 


### MEDICAL EXAMINER.

*A Semi-Monthly Journal of Medical Sciences.*

EDITED BY

N. S. DAVIS, M. D., AND F. H. DAVIS, M. D.

Chicago, Sept. 15th, 1873.

### EDITORIAL.

### CHICAGO MEDICAL COLLEGE.

The next regular College term in this institution will commence on Monday evening, October 6th, with an introductory lecture by N. S. Davis, M.D., Dean of the Faculty. The lecture will be in the College hall, corner of Prairie avenue and Twenty-sixth street, commencing at 7½ P.M. The coming term in the Chicago Medical College will open with a more complete and extended curriculum of instruction than any other medical school in this country. In addition to the three full didactic series, arranged to suit the progress of the student, and supplied with every needed means of illustration, the laboratories for individual study of practical chemistry and microscopy will be ample for the accommodation of all who may wish to enter them; while the Mercy Hospital and Free Dispensary will afford one general clinic daily, and six special clinics to small classes in as many different departments, embracing Gynecology, Ophthalmology and Otology, minor Surgery, Diseases of the Respiratory and Circulatory Organs, Cutaneous Diseases, and ordinary medical cases. The whole is reduced to a perfect system, and secures to the student, as a part of his regular course, those *special courses* of instruction which he cannot get in the Schools and Hospitals located either in the Eastern cities of our country or in those of Europe, without paying *extra fees* for the same.

